# Editorial for the Special Issue “Infrared Nanophotonics: Materials, Devices and Applications”

**DOI:** 10.3390/mi11090808

**Published:** 2020-08-26

**Authors:** Tadaaki Nagao

**Affiliations:** 1 International Center for Materials Nanoarchitectonics (MANA), National Institute for Materials Science, 1-1 Namiki, Tsukuba, Ibaraki 305-0044, Japan; NAGAO.Tadaaki@nims.go.jp; 2 Department of Condensed Matter Physics, Graduate School of Science, Hokkaido University, Kita 8, Nishi 5, Kita-ku, Sapporo 060-0810, Japan

Infrared light radiates from almost all the matter on earth and its strategic use will be an important issue for the enhancement of human life and the sustainable development of modern industry. Since it has frequency in the same region as phonons or molecular vibrations of materials, measuring its emission or absorption spectra helps us in characterizing and identifying the materials in a non-destructive manner. Meanwhile, if we can spectroscopically design the infrared emission by tuning the chemical composition or artificially controlling the nano- to mesoscale structures, it will have a great impact on industrial applications, such as in thermophotovoltaics, energy-saving drying furnaces, spectroscopic infrared light sources, and various types of infrared sensors.

In this Special Issue, recent studies by researchers who are working on MEMS-based infrared detectors, nanomaterial-based infrared detectors, thermal emitters, or fiber optics, have been contributed. Important topics of growing interests are the wavelength-selective infrared emitters and detectors where we can see rapid development in the field of nano-plasmonics and metamaterials, and we also collected contributions from narrow-band gap semiconductors.

This Special Issue collected 13 research papers, including one featured article. These are categorized as follows.

(1) Infrared nano/micro devices based on lithographic techniques and MEMS structures.

Dao et al. have demonstrated a compact design for membrane-supported, wavelength-selective infrared (IR) bolometers [1]. The fabricated devices exhibit a wide resonance tunability in the mid-wavelength IR atmospheric window by changing the size of the resonator of the devices, evidencing that the concept of the proposed wavelength-selective IR bolometers is realizable. Dao et al. also experimentally studied the dark-field scattering spectral mapping of plasmonic resonance from the free-standing Al bowtie antenna arrays and correlated their strong nearfield enhancement with the sensing capability by means of surface-enhanced Raman spectroscopy [2]. Doan et al. reported a quad-wavelength hybrid plasmonic–pyroelectric detector that exhibited spectrally selective infrared detection at four wavelengths—3.3, 3.7, 4.1, and 4.5 μm [3]. The narrowband detection was achieved by coupling the incident infrared light to the resonant modes of the four different plasmonic perfect absorbers based on an Al-disk-array placed on an Al_2_O_3_–Al bilayer, exhibiting great possibilities for miniature multi-wavelength spectroscopic devices. Yoshino et al. developed, a simple process to mechanically fabricate ordered Au nanodot arrays that respond to nearinfrared light, and also reported the feasibility of its application to plasmonic sensors [4]. The developed nanoprocess utilizes direct mechanical cutting of Au film by single-crystal diamond blades and further thermal processing to tune the Au nanodot shape and their plasmon resonance. Sakurai et al. studied a tungsten-SiO_2_-based metal insulator metal-structured metasurface for the thermal emitter of the thermophotovoltaic system [5]. The proposed emitter was fabricated by applying the photolithography method. The fabricated emitter has high emissivity in the visible to near-infrared region and shows excellent wavelength selectivity.

(2) Materials for infrared thermal emitters/absorbers and detectors based on compound semiconductors and their variants.

Ngo et al. reported the synthesis and demonstration of niobium-doped titanium dioxide for the application in plasmonic antenna and surface-enhanced infrared absorption [6]. The nanopatterns prepared by electron beam lithography, plasma etching/ashing processes showed well-defined antenna resonance as well as clear polarization/size dependence, which confirms that these materials are suitable for infrared plasmonic applications. Li et al. numerically studied the optical properties of hexagonal ITO nanodisk and nanohole arrays in the mid-infrared [7]. Field enhancement up to 10 times was observed in the simulated ITO nanostructures, and furthermore, they demonstrated the sensing of the surface phonon polariton from a 2-nm-thick SiO_2_ layer under the ITO disk arrays. Chiu et al. examined the optical properties of alloys with noble metals (Au and Pt). The six different metals (Ir, Mo, Ni, Pb, Ta, and W) which possess good properties for heat resistance, stability, and magnetism were mixed with noble metals to improve the properties [8]. The optical properties were calculated by density functional theory and they were used for further investigations of the optical responses of alloy nanorods. The results show that the studied alloy nanorods have wavelength-selective properties and can be useful for infrared devices and systems. Zhai et al. reported a mid-wave infrared (MWIR) and long-wave infrared (LWIR) dual-band photodetector capable of voltage-controllable detection band selection [9]. The voltage-tunable dual-band photodetector is based on multiple stacks of sub-monolayer quantum dots (QDs) and self-assembled QDs. By changing the photodetector bias voltages, one can set the detection band to be MWIR, or LWIR, or both, with high photodetectivity and low crosstalk between the bands.

(3) Infrared-sensing applications using fiber and laser technology, and hyperspectral camera.

Inada et al. evaluated the performance of a fluorescent detection system in an extirpated pig stomach and a freshly resected human stomach and were able to successfully detect NIR fluorescence emitted from the clip in the stomach through the stomach wall by the irradiation of excitation light (λ: 808 nm) [10]. The proposed combined NIR light-emitting clip and laparoscopic fluorescent detection system could be useful in clinical practice for accurately identifying the location of a primary gastric tumor during laparoscopic surgery. Chen et al. reported a sensor system composed of a quantum cascade laser (4.65 μm excitation wavelength), and a compact multiple reflection cell with a light path length of 12 m for sensitively detecting trace CO gas [11]. The sensor adopted the long optical path differential absorption spectroscopy technique (LOP-DAST) and obtained the minimum detection limit (MDL) of 108 ppbv by comparing the residual difference between the measured spectrum and the Voigt theoretical spectrum. Mu et al. studied tunable diode laser absorption spectroscopy (TDLAS) combined with wavelength modulation spectroscopy (WMS) using an interband cascade laser for detecting a trace amount of C_2_H_2_ [12]. The data show that the minimum detection limit is as low as 1 ppbv at an integration time of 63 s, and capable of detecting a variety of gases by changing the wavelength of the laser. Kim et al. reported a novel real-time remote temperature estimation method by applying a deep-learning-based regression method to midwave infrared hyperspectral images [13]. They proposed a method for real-time remote temperature measurement with high accuracy with the proposed surface-temperature, deep convolutional neural network and a hyperspectral thermal camera.

We would like to thank all authors for submitting their papers; most of them kindly contributed to this Special Issue in response to our invitation. We would also like to acknowledge all the reviewers for dedicating their time and timely reviews to improve the quality of this Special Issue. 
